# Teacher‐Led Universal Eating Disorder Prevention Programmes in Schools: A Scoping Review

**DOI:** 10.1002/erv.70064

**Published:** 2025-12-12

**Authors:** Jessica Parker, Lauren Makin, Karina Allen, Kate Tchanturia

**Affiliations:** ^1^ Department of Psychological Medicine King's College London Centre for Research in Eating and Weight Disorders Psychological Medicine London UK; ^2^ South London and Maudsley NHS Foundation Trust Eating Disorders Service London UK; ^3^ Department of Psychology Illia State University Tbilisi Georgia

**Keywords:** adolescence, eating disorders, prevention, school‐based programs, teacher‐led interventions

## Abstract

**Objective:**

This scoping review seeks to evaluate the efficacy of teacher‐led interventions in mitigating risk factors related to eating disorders, considering the necessity for universal prevention programmes that can be effectively administered by educators.

**Methods:**

A literature search was conducted via ERIC, PubMed, and Scopus, following PRISMA guidelines. This search focused on peer‐reviewed studies that evaluated teacher‐led programmes among adolescents aged 11–16. Data was extracted on study design, sample characteristics, intervention content, and outcomes. Quality appraisal was conducted using CASP and CONSORT to enable evaluation. Results were synthesised and guided by PRISMA.

**Results:**

From 16 studies, this review identified that teacher‐led programmes could effectively reduce risk factors, including thin‐ideal internalisation and body dissatisfaction. However, effectiveness requires an emphasis on teachers following program manuals, as deviations from the program often weaken outcomes, underscoring the need for comprehensive teacher training and support. The review also highlighted gender‐specific challenges, with some programmes being less effective for boys.

**Conclusions:**

Teacher‐led interventions are important for scalable and sustainable eating disorder prevention in schools; however, success depends on rigorous adherence to intervention protocols and ongoing support for educators. Future research should focus on long‐term efficacy, gender‐specific adaptations, and the comparative cost‐effectiveness of teacher‐led versus clinician‐led programmes.

## Highlights

- Teacher‐led programmes can lower eating disorder risks like thin‐ideal internalisation and body dissatisfaction, but their effectiveness relies on teacher adherence to guidelines and adequate training.
- The review highlighted gender‐specific challenges, noting that some programmes are less effective for boys, indicating a need for gender‐specific adaptations in the content and delivery of these interventions.
- Teacher‐led interventions may prevent eating disorders in schools, but more research is needed to compare their long‐term effectiveness and cost with clinician‐led programmes.

## 1 Introduction

Eating disorders are mental health conditions which have significant impacts on both physical and psychological health (Hambleton et al. 2022). As eating disorders often develop during adolescence and young adulthood, they influence educational and psychosocial functioning during critical developmental periods (Potterton et al. 2020). Globally, the number of 6 to 18‐year‐olds with eating disorders increased by 40% following COVID‐19, with Pastore et al. (2023) projecting worldwide rises in prevalence. In the UK, the incidence of eating disorders among female adolescents and young people has risen. Hospital admissions for teenagers with eating disorders increased by 59% following the first lockdown in 2020 (NHS England). Earlier identification and prevention are essential to minimise the impacts of eating disorders on so many young people and to reduce the strain on over‐stretched mental health services that are unable to provide timely treatment to the high volumes of patients (Eisler et al. 2022). This is compounded by the fact that many young people are presenting at services late on their journeys with more entrenched symptoms, which are difficult to treat, increasing relapse rates. Identifying young people earlier will produce more positive clinical outcomes (Brown et al. 2018).

Prevention strategies are categorised into three tiers based on target population: universal, selective, and indicated prevention (Han et al. 2025). Universal prevention target. all students, regardless of risk level and aim to promote positive body image and healthy eating attitudes schoolwide (Schlegl et al. 2025) Selective prevention targets higher risk individuals, such as those with body dissatisfaction, a family history, or exposure to harmful media messaging (Sarraj et al. 2025). Indicated prevention targets individuals demonstrating early symptoms of disordered eating (Schwartz et al. 2019). These programmes are more intensive and involve screening and tailored support.

Eating disorder prevention focuses on addressing key risk factors that arise before clinical symptoms develop, notably thin‐ideal internalisation, body dissatisfaction, and dieting behaviours (Downs 2025; Cimino et al. 2025; Hanson et al. 2025; Chan et al. 2025). Thin‐ideal internalisation involves aspiring to culturally promoted body shapes, usually a slim figure (Vankerckhoven et al. 2025). There is also growing concern around muscular‐ideal internalisation which is the drive for a lean and muscular body. This is linked to body dissatisfaction, compulsive exercise, supplement misuse, and disordered eating, particularly in males (Convertino et al. 2022; Stice and Shaw 2004). Media and cultural influences fuel both ideals and may require tailored, gender‐sensitive prevention strategies (Sicilia et al. 2023). These internalisations are closely associated with body dissatisfaction, which is a strong predictor of disordered eating across ages and genders (Byrne et al. 2024; Mora et al. 2022; Saul et al. 2022; Moyon et al. 2024; Moffitt et al. 2024). Dieting, especially when unsupervised or extreme, further increases risk, often leading to restrictive eating, bingeing, or purging behaviours (Cabaco et al. 2021; Ispas et al. 2025). Prevention programmes are designed to target these factors.

A range of prevention programmes have been developed and evaluated, each targeting different risk factors for body dissatisfaction and disordered eating. Cognitive dissonance‐based interventions, such as *The Body Project*, involve participants actively critiquing and rejecting societal appearance ideals—particularly the thin ideal—creating psychological discomfort (dissonance). This discomfort shifts attitudes and reduces the internalisation of harmful beauty standards, contributing to improved body image and reduced eating disorder risk.

Media literacy programmes, such as *Media Smart*, strengthen young people's ability to critically evaluate media messages that promote unrealistic or narrow beauty ideals. By increasing scepticism towards these messages and reducing the pressure to conform to appearance‐based norms, such programmes have been shown to enhance body satisfaction and resilience against media influence. Both approaches address key sociocultural influences and can be valuable components of school‐based prevention strategies. Becker and Stice et al. (2017) found that the *Body Project* intervention significantly reduced eating disorder risk factors and symptoms, and also demonstrated that it lowered the future onset of eating disorders among adolescent girls and young women. Mindfulness‐based media literacy programmes like *MAiSTEP* not only help people critically evaluate media messages and enhance body satisfaction, but also cultivate present‐moment awareness, emotional regulation and non‐judgemental outlooks (Wilksch and Wade 2014; Buerger et al. 2019). Furthermore, digital interventions, *Student Bodies* (Celio et al. 2000) and large‐scale programmes like *Dove Confident Me* (Diedrichs et al. 2021) offer scalable, technology‐driven resources that promote body confidence and self‐worth through interactive, often self‐guided content. These universal programmes are particularly suited to school settings where flexibility and accessibility are crucial. This inclusive approach ensures that protective factors including media literacy, emotional regulation, and positive body image, are nurtured across the school.

In England, school‐based mental health provision advanced through increased government focus, policy development, and funding. This began with the 2010–2015 mental health service reform policy, aiming to give mental and physical health equal priority (Department of Health and Social Care and Department for Education 2017), leading to the 2017 *Transforming Children and Young People's Mental Health* Green Paper. This introduced Mental Health Support Teams to work alongside designated Senior Mental Health Leads (Department for Education 2016).

The Department for Education (2015), collaborating with Public Health England, developed a whole‐school framework built around eight principles. Implementing these requires coordinated action and shared responsibility among staff, with senior leadership playing a critical role in driving change. Their support is essential for embedding eating disorder prevention into the wider school culture — ensuring it is not limited to classroom content but reflected in a broader ethos that promotes body neutrality (Pastore et al. 2023; NHS England).

Health and well‐being can be supported through a whole‐school approach with staff training being crucial for identifying and addressing concerns, including eating disorders (Koreshe et al. 2023; Lekamge et al. 2025). To ensure efforts are effective, schools must monitor impact by formally assessing young people's needs using standardised outcome measures to inform decision‐making of which a broad array is available. Many schools utilise well‐validated instruments such as the *Strengths and Difficulties Questionnaire* (Goodman 2001), the *Warwick‐Edinburgh Mental Wellbeing Scale* (Tennant et al. 2007), and the *Paediatric Symptom Checklist* (Jellinek et al. 1999). Recently, the *Mental Health in Schools Questionnaire (MHISQ)* has demonstrated strong potential across mainstream and inclusive educational settings (Francis 2025), enabling schools to holistically monitor the well‐being of a student population.

Universal screening is becoming increasingly common, with many schools now employing multi‐gate models; beginning with brief, broad assessments and providing more targeted follow‐up for those who require it (Palmer et al. 2025). These approaches are being backed by policy, not least because they enable schools to identify concerns early and respond more effectively. UK guidance including CORC (Child Outcomes Research Consortium [CORC] 2024), encourages schools to combine subjective well‐being data with practical indicators, such as attendance and academic progress. Nevertheless, recent research highlights ongoing barriers, including limited capacity and resources (Spencer et al. 2025).

Furthermore, working with families and carers is fundamental, as they significantly shape young people's mental health especially through messages they convey about diet, exercise, and self‐worth (Winter et al. 2025). Schools are well‐positioned to play key roles in family/carer psychoeducation, ensuring that support for mental health is consistent across home and school environments. As Pursey et al. (2022) notes, school professionals often lack the confidence and training to engage effectively with families around eating disorders, underscoring the need for greater emphasis on this within whole‐school approaches. This includes supporting schools to identify signs, to work preventatively by increasing awareness in both young people and their families, and to adapt the school day and broader aspects of the education experience for young people who may have receive a diagnosis. These areas of focus will only be effective when supported by clear and comprehensive training which consider the complexities of working in schools. School‐specific complexities include ever‐increasing curriculum demands and competing time pressures (Schäfer et al. 2025; March et al. 2022).

Prevention programmes delivered in schools follow one of three models: teacher‐led, clinician‐led, or peer‐led. Teacher‐led programmes are delivered by trained educators within schools (Atkinson et al. 2024), clinician‐led approaches involve health professionals delivering sessions directly. Peer‐led models engage trained students to deliver content to their peers, fostering relatability and shared experience (Patmore and Ranzenhofer 2024). Few studies consider the effective implementation of teacher‐led universal programmes for eating disorders, specifically in English schools, mainly where teachers are the facilitators (Sharpe et al. 2013). Knightsmith et al. (2014) identified a lack of knowledge and confidence teaching about eating disorders evidenced by the finding that many young people had not previously received any lessons but had experienced some positive interactions with their teachers when they required support.

The rising prevalence of adolescent eating disorders highlights an urgent need for collaborative efforts between healthcare services and the education sector to enhance awareness, early identification, and prevention strategies in UK secondary schools (Ward et al. 2025). Schools, as central hubs in young people's lives, can implement non‐specialist prevention and identification programmes, especially given the strain on overburdened and under‐resourced health services. With appropriate support and training, teachers can be pivotal in delivering universal programmes, addressing eating disorders rates, and mitigating their long‐term impact on well‐being (Elms and Higgins 2022).

### 1.1 Objectives

Given the need for eating disorder prevention programmes that teachers can effectively implement, this scoping review aimed to:

- Assess the effectiveness of teacher‐led interventions in reducing risk factors associated with eating disorders.
- Examine the impact of teacher adherence to intervention protocols on the efficacy of these programmes.

## 2 Methods

### 2.1 Search Strategy

A scoping review methodology was selected in line with guidance from Arksey and O'Malley (2005), Levac et al. (2010), and Munn et al. (2018), who recommend this approach when the goal is to explore the breadth of literature, map key concepts, and identify research gaps rather than assess the effectiveness of interventions in a narrowly defined manner.

**FIGURE 1:**
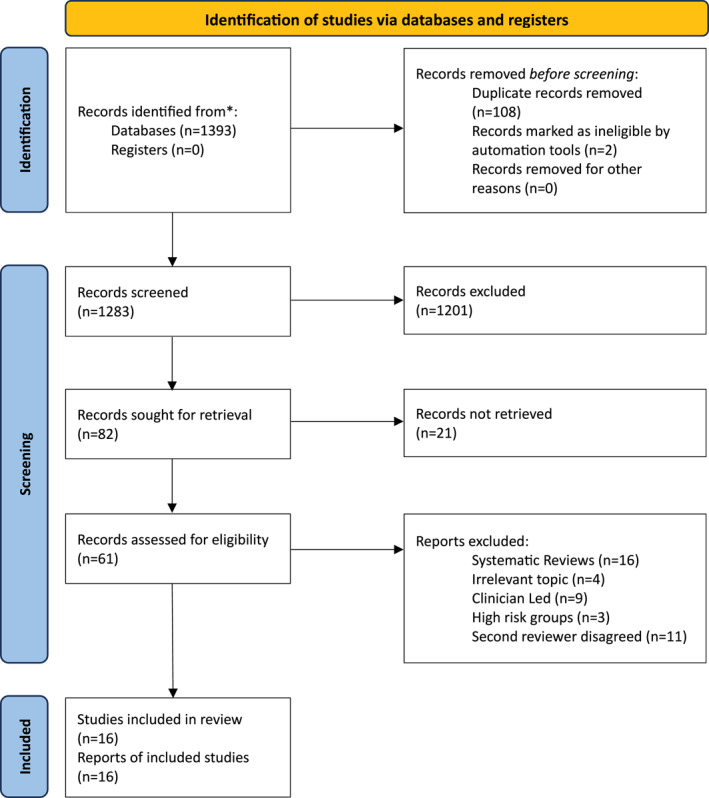
PRISMA flowchart.

This scoping review was conducted in September 2024 following Preferred Reporting Items for Systematic Reviews and Meta‐Analyses (PRISMA) guidelines (Figure 1) to promote replication.

**FIGURE 2:**
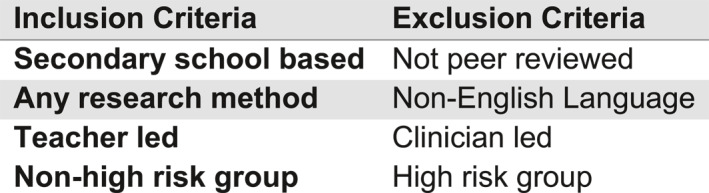
Inclusion and exclusion criteria.

The search identified peer‐reviewed articles that investigated the effectiveness and implementation of school‐based eating disorder prevention programmes, conducted by teachers/education professionals. This process utilised ERIC, PubMed and Scopus bibliographic databases via a dual‐pronged approach. Initially, a broad search was used to derive all results related to ‘school‐based eating disorder prevention.’ To streamline the search, the keywords and subject‐specific terms used included ‘eating disorder prevention,’ ‘school‐based,’ ‘adolescents,’ ‘eating disorders and schools,’ ‘body image,’ and ‘disordered eating’. These were used in various combinations to ensure comprehensive literature retrieval. The search was limited to studies published in English and secondary school‐aged children. However, the terms ‘high school’, ‘middle school’, and ‘senior school’ were also used to include other relevant English language papers. Their similar education systems ensured that the search algorithm generated all relevant articles from different countries, including Australia, the USA, New Zealand and Canada which included schools that cover the same student age range as English secondary schools. All schools were included regardless of whether they were fee‐paying or state funded, and school selection criteria were disregarded. Duplicate articles were removed, and titles and abstracts were independently screened by JP and LM for relevance to the research question. Full‐text articles were then assessed for eligibility based on predefined inclusion and exclusion criteria (Figure 2), focusing on studies providing empirical evidence on outcomes of teacher‐led eating disorder prevention programmes.

### 2.2 Article Inclusion

An article was deemed to be relevant for inclusion if it adhered to the following parameters: (i) utilised samples of education professionals working with children or young people of ages 11–18 (this recognises the secondary and high school ages within the Anglosphere); (ii) both quantitative and qualitative studies; (iii) the interventions were teacher/education professional led or facilitated; (iv) published in English between 2000 and 2024. This timeframe was chosen to capture both foundational and contemporary interventions relevant to the current UK educational and policy landscape.

Furthermore, the relevant articles need to have been published in a peer‐reviewed journal. ERIC, PubMed and the Scopus bibliographic databases categorised these journals.

### 2.3 Article Exclusion

The exclusion criteria framework consisted of (i) articles not peer‐reviewed due to concerns about undetected methodological flaws and uncertainty around credibility and reliability of the findings; (ii) clinician‐led interventions and peer‐led interventions, as this review's purpose is to ascertain teacher‐led interventions; (iii) high‐risk groups such as sports academies, alternative provision, and ballet schools as these may not represent the general school population which is intended for this paper.

In addition, 16 meta‐analyses and reviews were excluded to avoid duplication and focus on mapping original research.

#### 2.3.1 Rationale for Exclusion of Previous Reviews

Despite growing recognition of adolescent eating disorders as a major public health concern, teacher‐led universal prevention programmes remain significantly under‐explored and inconsistently implemented, particularly within UK schools. While meta‐analyses (Stice et al. 2007, 2021; Stice and Shaw 2004) and systematic reviews (Ciao et al. 2014; Watson et al. 2016; Wong et al. 2024) have established that prevention programmes can reduce risk factors such as thin‐ideal internalisation, body dissatisfaction, and dieting, these reviews also indicate that universal approaches often yield smaller effects than selective or indicated interventions. Furthermore, although digital and e‐mental health tools (Linardon et al. 2020) offer promising scalability, they are frequently associated with high dropout rates, limited user‐centred design, and a lack of direct comparison with in‐person delivery. Several studies (Grave 2003; Le et al. 2017; Leme et al. 2018) reinforce that universal programmes often fall short in changing actual behaviours unless they are interactive, theory‐driven, and developmentally tailored. Reviews (Koreshe et al. 2023; Yager and O'Dea 2005; Yager et al. 2013) further highlight that, while teachers and peer educators are well‐positioned to deliver such interventions, barriers related to confidence, training, and practical support persist. Additionally, issues of cultural, gender, and socioeconomic inclusivity are frequently overlooked (Nicula et al. 2022), and opportunities for integrating early detection and mental health literacy remain largely untapped (Kalindjian et al. 2022).

This scoping review addresses these critical gaps by systematically mapping the current landscape of teacher‐led universal eating disorder prevention programmes, evaluating their feasibility, inclusivity, and outcomes, and generating evidence to support more effective, equitable, and sustainable implementation in school settings. In doing so, it provides a comprehensive synthesis that extends beyond the scope of previous reviews, offering new insights into the practical challenges and opportunities associated with teacher‐led approaches.

### 2.4 Quality Appraisal Process

The quality of these articles was analysed per the protocol set out by the Critical Appraisal Skills Programme (Critical Appraisal Skills Programme UK), as presented in Table 1. This tool was selected as it provides a comprehensive framework to appraise the studies included to enhance the transparency of this scoping review. Non‐randomised, uncontrolled feasibility was appraised utilising Eldridge et al. (2016) extension to CONSORT for randomised pilot and feasibility trials.

**TABLE 1:** CASP: Randomised control study.

Questions	Atkinson et al. (2024)	Austin et al. (2007)	Canetti et al. (2009)	Favaro et al. (2005)	Kristoffersen et al. (2022)	O’Dea and Abraham (2000)	Phelps et al. (2000)	Schmidt et al. (2017)	Sharpe et al. (2013)	Stewart et al. (2022)	Wade et al. (2003)	Warschburger and Zitzmann (2018)	Wolter et al. (2021)	Yager et al. (2023)
Did the study address a clearly focused issue?	Yes	Yes	Yes	Yes	Yes	Yes	Yes	Yes	Yes	Yes	Yes	Yes	Yes	Yes
Was the assignment of participants to interventions randomised?	Yes	Yes	Yes	Yes	Yes	Yes	Yes	Yes	Yes	Yes	Yes	Yes	Yes	Yes
Were all participants who entered the study accounted for at its conclusion?	Yes	Yes	Yes	Yes	Yes	Yes	Can’t tell	Yes	Yes	Yes	Yes	Yes	Yes	Yes
Were the participants ‘blind’ to intervention they were given? were the investigators ‘blind’ to the intervention they were giving to participants? were the people assessing/analysing outcome/s ‘blinded’?	No	No	No	Yes	No	No	No	No	No	No	No	No	Partial blinding	No
Were the study groups similar at the start of the randomised controlled trial?	Yes	Yes	Yes	Yes	Yes	Yes	Can’t tell	Yes	Yes	Yes	Yes	Yes	Yes	Yes
Apart from the experimental intervention, did each study group receive the same level of care (that is, were they treated equally)?	Yes	Yes	Yes	Yes	Yes	Yes	Yes	Yes	Yes	Yes	Yes	Yes	Yes	Yes
Were the effects of intervention reported comprehensively?	Yes	Yes	Yes	Yes	Yes	Yes	Yes	Yes	Yes	Yes	Yes	Yes	Yes	Yes
Was the precision of the estimate of the intervention or treatment effect reported?	Yes	Yes	Yes	Yes	Assessment of acceptability was the focus not precision.	Yes	Yes	Yes	Yes	Yes	Yes	Yes	Yes	Yes
Do the benefits of the experimental intervention outweigh the harms and costs?	Yes	Yes	Yes	Yes	Yes	Yes	Yes	Yes	Yes	Yes	Yes	Yes	Yes	Yes
Can the results be applied to your local population/in your context?	Yes	Yes	Yes	Yes	Yes	Yes	Yes	Yes	Yes	Yes	Yes	Yes	Yes	Yes
Score out of 10	9/10	9/10	9/10	9/10	9/10	9/10	7/10	9/10	9/10	9/10	9/10	9/10	9/10	9/10

CASP: Cohort study		
Questions	Berger et al. (2014)	Forbes et al. (2023)
Did the study address a clearly focused issue?	Yes	Yes
Was the cohort recruited in an acceptable way?	Yes	Yes
Was the exposure accurately measured to minimise bias?	Yes	Yes
Was the outcome accurately measured to minimise bias?	Yes	Yes
Have the authors identified all important confounding factors?	Moderately	Yes
Have they taken account of the confounding factors in the design and/or analysis?	Yes	Yes
Was the follow up of subjects complete enough?	Yes	Yes
What are the results of this study?	Girls body self‐esteem improved as did their eating behaviour. Effects were less in boys.	There was a positive impact on body image.
How precise are the results?	Statistically significant results are clearly presented.	Confidence intervals provided.
Do you believe the results?	Yes	Yes
Can the results be applied to the local population?	Yes, but with similar schools and cultures otherwise cultural adaptations will be required.	Yes, but only same type of setting.
Do the results of this study fit with other available evidence?	Yes	Yes
What are the implications of this study for practice?	Teacher‐led programs can be cost‐effective and contribute towards improvements in eating behaviours.	It is expected that the program could make a meaningful difference in other similar settings.
Score out of 13	12/13	12/13

EndNote was utilised to manage the search results for article management and organisation, allowing citation tracking, duplication removal, and reference organisation. This facilitated a streamlined review process, ensuring accuracy in selection and citation of studies. This critical appraisal aided identification of the methodological quality of each study, thereby ensuring that conclusions drawn from the scoping review were based on high‐quality evidence. The PRISMA flow diagram was prepared (Figure 1) to represent the study selection process visually, detailing the number of records identified, screened, assessed for eligibility, and included in the review, providing a transparent and replicable methodology.

## 3 Results

The scoping review identified 1393 articles. After screening these articles based on the titles and abstracts, full texts were sourced. In accordance with PRISMA protocol, 16 systematic reviews were subsequently excluded. A further 27 articles were excluded for reasons which included high‐risk samples, irrelevant topics, the involvement of clinicians or researchers to deliver the interventions in person, or if they did not specify who delivered the intervention (Figure 1). Additionally, coauthor LM also scored selected articles to ensure reliability and to verify documents fit the inclusion criteria. LM checked the consistency of the criteria application. Any articles that LM did not agree met the criteria were also excluded.

### 3.1 Characteristics of Included Studies

Within this scoping review, the majority of samples comprised of adolescents aged 11–16 years, with some spanning this age and beyond (up to 25 years). These studies were included as some of their participants still fell within the inclusion criteria. Many studies did include mixed‐gender samples (46, 74, 77, 80, 83, 84; Table 2). However, some were limited to females only (75, 76, 78, 82, 85; Table 2) and one to males only (Yager et al. 2023). The settings were international, including USA (Austin et al. 2007), Australia (Kristoffersen et al. 2022; Wade et al. 2003; Yager et al. 2023; Forbes et al. 2023), Germany (Berger et al. 2014; Warschburger and Zitzmann 2018; Wolter et al. 2021), Israel (Canetti et al. 2009), Italy (Favaro et al. 2005) and the UK (Atkinson et al. 2024; Sharpe et al. 2013; Eldridge et al. 2016; Stewart et al. 2022). No studies specified the ethnicity or socioeconomic status of the student samples, and only one stated race (1; Table 2). Most studies were delivered by educators; however, a small number involved adaptations from standard teacher‐led formats. For example, some studies employed teacher‐facilitated video‐based delivery (Stice et al. 2021), used externally developed materials (Forbes et al. 2023), or examined universal adaptations of previously selective interventions (Atkinson et al. 2024). One study (Yager et al. 2023) involved delivery structures with less detailed reporting on teacher facilitation. These were retained as all programmes were implemented within classroom settings under educator oversight, aligning with our operational definition of teacher‐led implementation.

**TABLE 2:** Study demographics.

Study/Author	Age group	Sex assigned at birth and/or gender	Race	Ethnicity	Socioeconomic status	Location	Participants
Atkinson study	13–15 years	Boys and girls	Primarily White	Not specified	Not specified	South Wales and South‐West England	Boys and girls, mostly UK‐born, primarily White, English‐speaking
Austin (5‐2‐1 go!) study	11–13 years	Girls and boys	Not specified	Not specified	Not specified	Massachusetts, USA	1451 students (749 girls, 702 boys), assessing disordered weight‐control behaviours
Berger study	12–17 years	Female students	Not specified	Not specified	Not specified	Switzerland	Female students, focusing on cognitive‐behavioural therapy (CBT) for eating disorder prevention
Canetti study	14–19 years	Male and female	Not specified	Not specified	Not specified	Israel	Both male and female participants, focusing on eating disorders, body image, and media influences
Favaro study	12–14 years (students)	Mixed‐gender students, teachers	Not specified	Not specified	Not specified	Italy	Teachers and students, focusing on eating disorder prevention through teacher engagement
Forbes study	18–24 years	Male and female	Not specified	Not specified	Not specified	Australia	Both male and female students, focusing on body dissatisfaction and media influences
Kristofferson study	14–20 years	Male and female	Not specified	Not specified	Not specified	Norway	Both male and female participants, focusing on the impact of social media on body image
O'Dea and Abraham study	11–14 years	Mixed‐gender	Not specified	Not specified	Not specified	Australia	Mixed‐gender students, focusing on body image, eating attitudes, and behaviour interventions
Phelps study	11–25 years	Female students	Not specified	Not specified	Not specified	USA	Female students, focusing on eating disorder prevention across different age groups
Sharpe study	12–14 years	Female students	Not specified	Not specified	Not specified	UK (London and East England)	Female students aged 12–14 from diverse ethnic and socioeconomic backgrounds in UK all‐girls schools
Schmidt study	14–20 years	Mixed‐gender	Not specified	Not specified	Not specified	Germany	Mixed‐gender participants, focusing on an eating disorder prevention program
Stewart study	18–25 years	Female students	Not specified	Not specified	Not specified	USA	Female students, focusing on body dissatisfaction and eating disorder prevention
Wade study	15–17 years	Mixed‐gender	Not specified	Not specified	Not specified	Australia	Mixed‐gender students, focusing on media literacy and eating disorder prevention
Warschburger study	11–15 years	Male and female	Not specified	Not specified	Not specified	Germany	Both male and female students, focusing on body image and disordered eating behaviours
Wolter study	13–17 years	Male and female	Not specified	Not specified	Not specified	Switzerland	Both male and female students, examining the effectiveness of a prevention program
Yager study	11–18 years	Mixed‐gender	Not specified	Not specified	Not specified	Australia	Mixed‐gender students, focusing on a media literacy program to prevent eating disorders

### 3.2 Quality Appraisal

All studies were assessed using the CASP tools for Randomised Control Studies or for cohort studies. Studies were generally deemed to be of high quality, with most randomised control studies being 9/10. Many of the studies within this area are limited due to the lack of blinding as a control which may have introduced bias (Table 1). Cohort studies ranged between 11 and 12 for quality, and this can be attributed to their methodological rigour and acknowledgement of limitations where these existed. They were found to have promising outcomes but need randomised control trials with a larger sample size and a control condition (Table 1).

### 3.3 Synthesis of Results

#### 3.3.1 Effectiveness of Teacher‐Led Programmes on Body Image and Self‐Esteem

The results illustrate that positive outcomes are possible, particularly when programmes are delivered with high fidelity and structure. For instance, O'Dea & Abraham (2000) (O’Dea and Abraham 2000) demonstrated improved body satisfaction and self‐esteem scores that were sustained at 1‐year follow‐up (Table 3). (Sharpe et al. 2013) found that a teacher‐led body image programme significantly improved body esteem and reduced thin‐ideal internalisation, with effects maintained at 3 months, although the study was underpowered at follow‐up. Kristoffersen et al. (2022) also reported modest improvements in body appreciation and media literacy, using teacher‐facilitated video‐based content. Stewart et al. (2022) found that teacher‐led programmes were less effective than clinician‐led ones in improving body satisfaction, highlighting the potential importance of training and programme fidelity.

**TABLE 3:** A summary of scoping review findings.

Study	Aim	Design	Sample	Outcome measures	Follow‐up time points	Main findings	Methodological strengths (S) limitations (L)
Atkinson et al. (2024)	To investigate the acceptability and feasibility of two teacher led, universal eating disorder programs	Mixed method randomised control Trial	A total of 288 students attending UK schools (50.69% boys, 48.96% girls, 0.35% other gender) aged 13–15 years took part. Six teachers from the two intervention schools also participated across several subjects' area.	Primary outcomes: Body esteem, body satisfaction, weight and shape concerns, and positive and negative affect. Secondary outcomes assessed: Emotion regulation difficulty, overall well‐being, life disengagement due to appearance concerns, mindfulness, and internalisation of appearance ideals.	Follow‐up assessments were conducted at post‐intervention (6 weeks) and at 2 months after the intervention.	Teacher‐led mindfulness‐based (MBI) and dissonance‐based (DBI) interventions were found to have a moderate acceptance level amongst students and teachers.	S: The interventions were well‐structured with specific lesson plans, visual aids, and interactive activities and the acceptability and feasibility was assessed via a mixed method approach.
						Improvements in body esteem from those receiving DBI were demonstrated (Cohen's *d* = 0.34). MBI resulted in increased positive affect (Cohen's *d* = 0.58). Effects were sustained at follow up.	L: The follow‐up period of only 2 months post‐intervention may not have been sufficient to capture the long‐term impacts of the interventions.
						High adherence to programme protocol: The programme was delivered using structured lesson plans, visual aids, and interactive activities. Teachers followed the protocol closely, and positive effects were sustained at follow‐up.	L: The lack of ethnic and cultural diversity in the sample means the interventions' effectiveness in broader contexts remains uncertain.
Austin et al. (2007)	To assess the effect of a school‐based program aimed at encouraging healthy eating habits and regular physical exercise on disordered weight management behaviours among pre‐teen girls and boys.	Quantitative randomised control Trial	749 girls and 702 boys aged 11–13 at baseline attending schools in Massachusetts, USA	Primary outcome: Incidence of disordered weight‐control behaviours (self‐induced vomiting, laxative or diet pill use in last 30 days).	Follow‐up: After two school years	The intervention reduced new disordered weight‐control behaviour in girls significantly. After 2 school years, 1.2% in intervention schools versus 3.6% in control reported such behaviours. Rates were two‐thirds lower in intervention girls (OR: 0.33, 95% CI: 0.11–0.97), who also remained stable in subsequent analysis. No significant effect was seen in boys.	S: A randomised control trial design enhanced the reliability and validity of findings. Multivariate analyses were also used, providing control over factors including sex, heightening the accuracy of conclusions drawn.
						Modereate adherence to programme protocol:	L: Large sample size but limited to 13 schools in Massachusetts which may not be generalisable to other populations.
						The programme structure was generally followed. There were no major deviations noted, although reporting on adherence was limited.	L: Self‐reported data which was gathered in schools. Therefore, under or over reporting could have impacted on the reliability of the findings.
Berger et al. (2014)	To assess the effects of the Torera prevention programme	Mixed methods (telephone interview, questionnaires)	533 girls and boys with a mean age of 13.1 from schools in Thuringia, Germany. Experimental group: *N* = 188 and control group *N* = 345.	Primary outcomes: Conspicuous eating behaviour (SCOFF, EAT‐26D) and body self‐esteem (FBeK).	Baseline (6th grade) and post‐intervention (7th grade), interval 189–616 days (average 394).	Girls displayed small to moderate improvements with an increased body esteem level and reduced eating related risk taking (*d* = 0.35–0.66).	S: The low implementation costs make this a viable model for schools in the UK who have finite budgets.
						Effects were limited for boys with only a small effect size in their eating attitudes (*d* = 0.35).	L: The lower effects for boys mean that further adaptations to the programme may be required to ensure that it is also useful for boys.
						It was found to be a cost‐effective program which can be added into the existing curriculum with minimal work involved for teachers.	L: The follow‐up period varied greatly (189–616 days), which might impact the consistency of long‐term outcomes and the assessment of the intervention's sustained effects.
						Moderate adherence to programme protocol: The programme was implemented broadly and incorporated into the curriculum. However, follow‐up times varied and detailed fidelity checks were not always reported.	
Canetti et al. (2009)	The aim of this study was to evaluate whether a structured primary prevention program could influence the eating‐related attitudes of middle‐school female students. It is part of a longitudinal follow‐up designed to assess the effectiveness of primary prevention in addressing maladaptive eating behaviours during adolescence.	This study is part of a long‐term follow‐up study	The study was conducted between 2000 and 2003 and involved 330 female 7th‐grade students from a suburban middle school in central Israel. Classes were randomly assigned to either the experimental or control group over 4 years. Each year, four of the nine 7th‐grade classes were assigned to the experimental group, and five to the control group. Thirty experimental students and 10 control students were excluded due to refusal to participate. The experimental group included 231 students, and the control group included 59 students, each attending at least five of six sessions. Additionally, 103 experimental and 37 control students from 2001 to 2002 were re‐analysed after 1 year.	Primary outcome: Eating‐related attitudes measured by the Treatment efficacy scale (TES). Secondary outcomes: Specific TES domains, including media influence, body image perceptions, attitudes toward dieting, and exercise.	Post‐intervention and at 1 year	There was a significant improvement in the total score of the Treatment efficacy scale (TES) from baseline to post‐intervention (1.94 ± 0.28 to 2.05 ± 0.32, t (230) = 4.78,*p* < 0.001	S: Utilising a prospective, longitudinal approach allows for the observation of sustained effects over time, providing robust data on the long‐term impact of program on eating‐related attitudes. Additionally, using a control group helps to establish a causal relationship between the intervention and observed changes in attitudes.
						After 1 year, the experimental group (103 students) maintained significant improvements in the total TES score (1.89 ± 0.28 to 2.07 ± 0.27, t (102) = 5.66,*p* < 0.001 and in several specific items, indicating sustained positive changes. The control group also showed some improvement. At 1‐year follow‐up, but the changes were less significant than those in the experimental group.	L: The study was conducted in one school which limits the generalisability of the findings to other schools.
						Low adherence to programme protocol: The study did not clearly report the degree of teacher adherence to the intervention protocol, raising questions about delivery consistency.	L: There was a high attrition rate of around 50% at the 1‐year follow‐up, which could bias the results with regards to the long‐term impacts.
Favaro et al. 2005	The aims were to evaluate the effectiveness of a prevention program implemented by teachers specifically trained for this role. To observe the response to the intervention, particularly among low‐risk girls.	A randomised control design was used. Participants were assessed at baseline and during a 1‐year follow‐up using clinical interviews and the eating attitudes Test (EAT‐40).	Nine classes consisting of 141 girls from a vocational training school in Mestre, near Venice, were selected. At the baseline assessment, 138 girls (98%) participated, and 132 (including three who missed the baseline) participated at the 1‐year follow‐up. Overall, 129 girls were assessed at both time points, resulting in a 91.5% participation rate. The girls were aged 16–18 years (mean age = 17.0 ± 1.1). Three classes were randomly assigned to the intervention group and six to the control group, with randomization done at the class level to account for intraclass response dependence. Control group subjects were analysed as a single class.	Primary outcome: Development of full or partial eating disorders assessed by SCID and EAT‐40. Secondary outcomes: Bulimic symptoms (EAT Bulimia subscale), strict dieting, fasting, unhealthy weight control behaviours, and importance of body weight/shape for self‐esteem.	1 year	Bulimia subscale scores of the eating attitudes Test (EAT) for the intervention group had an effect size of 0.55. The control group demonstrated no change in these scores. This difference was statistically significant (ANOVA for repeated measures, F (1,125) = 8.46, *p* < 0.005).	S: Trained psychologists and psychiatrists conducted a standardised clinical Interview (SCID) which ensures an objective assessment of the intervention's usefulness.
						No significant differences in BMI or total EAT scores were identified between the prevention and control groups. BMI: Prevention group ‐ 20.8 ± 2.6 (baseline), 20.9 ± 2.7 (follow‐up) compared with the control group ‐ 20.6 ± 2.4 (baseline), 20.8 ± 2.3 (follow‐up). ANOVA F (1,125) = 0.06, NS.	L: Changes in broader psychological factors such as self‐esteem and anxiety which are frequently comorbid with eating disorders were not considered.
						Total EAT scores: Prevention group ‐ 18.6 ± 10.3 (baseline), 15.8 ± 9.9 (follow‐up) compared with the control group ‐ 18.0 ± 13.0 (baseline), 15.2 ± 12.4 (follow‐up). ANOVA F (1,125) = 0.00, NS.	L: The extent to which the teachers' delivery of the intervention adhered to the protocol was not reported on. Any variations could impact how the study was received.
						Low adherence to programme protocol: No adherence data was provided. It is possible that variations in how teachers delivered the programme could have influenced the results.	
Forbes et al. (2023)	This research aimed to examine whether the universal co‐educational prevention program designed for audiences in the United Kingdom (Dove confident Me, DCM) was both an acceptable and effective intervention when delivered by teachers to adolescent girls in a single‐sex Australian school.	A quasi‐experimental design to replicate the Dove confident Me (DCM) body image program. Study 1 evaluated the original DCM program among Grade 8 girls and compared the outcomes with a matched comparison group from three other schools. Study 2 introduced minor adaptations to the program before testing it for its suitability within the Australian education context. Data were collected at three stages: Pre‐test, post‐test, and during a follow‐up after 3 months, offering an insight into longer term impacts. A combination of validated self‐reported measures were used.	198 Grade 8 girls from a single‐sex Australian school who participated in the Dove confident Me program were included. The comparison group comprised of 208 girls from a further three schools.	Primary outcomes: Body esteem, body appreciation, self‐esteem, and future plans.	Post‐intervention and at 3 months	Both the intervention and comparison groups showed significant improvements in self‐esteem, body‐esteem, and body appreciation over time. Specifically, self‐esteem increased with a mean difference of +0.48 (*p* < 0.001), body‐esteem improved with a mean difference of +0.44 (*p* < 0.001), and body appreciation increased with a mean difference of +0.42 (*p* < 0.001) across both groups.	S: This study highlights the need to adapt school‐based programs to fit the context of the children and young people access it.
						Significant reductions in the internalisation of the thin‐ideal (mean difference of −0.53, *p* < 0.001), perceived sociocultural pressure (mean difference of −0.37, *p* < 0.001), social comparison (mean difference of −0.31, *p* < 0.001), and dietary restraint (mean difference of −0.29, *p* < 0.01) across both groups.	
						There were no significant differences between the intervention and comparison groups in any of these measures, indicating that the Dove confident Me program did not have a distinct impact compared to the control group.	L: The 3‐month follow‐up period may have been too short to fully assess the long‐term impact of the intervention on body image outcomes.
				Secondary outcomes: Internalisation of appearance ideals, sociocultural pressures, social comparisons, appearance conversations, dietary restraint, perceived maternal pressure, and program acceptability.		Participants rated the program highly for comfort (mean rating of 4.3 out of 5), importance (4.2 out of 5), and teacher effectiveness (4.1 out of 5), but gave lower ratings for enjoyment (3.0 out of 5) and helpfulness (3.2 out of 5).	
						Teachers reported high confidence in program delivery (mean confidence rating of 4.4 out of 5) but commented that some students were not engaged during the lessons. This reduced the teachers' perceptions of program usefulness.	L: Student only experienced moderate enjoyment helpfulness, which could suggest the need for further adaptation.
						Moderate adherence to programme protocol: Teachers reported delivering the programme with confidence and mostly adhered to the content. However, some variation in student engagement and lesson delivery was noted.	
Kristoffersen et al. (2022)	Modify the micro interventions developed by Atkinson and Diedrichs (Celio et al. 2000) through collaborative consultation with students and teachers.	Mixed‐methods design via a person‐based approach and a randomised feasibility trial.	*N* = 5 school staff from a private school in Southern Australia reviewed the delivery materials.	Primary outcomes: State risk factors (weight satisfaction/distress, appearance satisfaction/distress, appearance‐ideal internalisation, media pressures, mood) and trait measures (weight and shape concerns, body appreciation, sociocultural pressures, affect, self‐compassion, self‐criticism).	Immediate post‐intervention and at 1 week.	The study had a participation rate of 44.8% with a retention rate of 84.2% at the 1‐week follow‐up. These figures suggest that the intervention was feasible within the school setting.	S: The study gained ongoing feedback from both students and staff to refine and adjust the intervention materials, ensuring they were more closely aligned with the specific needs and preferences of the target audience, thereby increasing the interventions' relevance and potential for effectiveness.
	Evaluate the feasibility of implementing these interventions in secondary schools and identify the research methods required for broader large‐scale evaluation.		*N* = 15 male and female students' participants in the focus groups to review materials.			Acceptability ratings indicated that the self‐compassion intervention had higher acceptability among girls, with 100% of girls in this group stating they would recommend it to a friend. In contrast, boys rated the cognitive‐dissonance intervention higher in some aspects, such as lesson endorsement but they generally found the self‐compassion intervention more comprehensible.	
	Measure the acceptability of the interventions among students.		*N* = 101 students aged 15 to 17 received either a cognitive dissonance or self‐compassion‐based intervention (cognitive‐dissonance intervention: 27 girls, 18 boys, 1 non‐binary). (Self‐compassion intervention: 27 girls and 28 boys).			Both interventions showed favourable changes in state outcomes, such as weight and appearance satisfaction. However, boys receiving cognitive‐dissonance intervention experienced a notable decrease in positive mood, despite positive changes in other areas.	L: Although the study employed pre‐ and post‐ assessments to evaluate changes in outcomes, the absence of a control group limits the ability to establish a direct causal relationship between the interventions and the observed effects. It is challenging to determine whether the changes were truly due to the interventions or influenced by other external factors.
	Investigate within‐group changes in both state and trait outcomes, by gender, to gauge the direction of any changes, identify any potential harmful effects, and refine the feasibility of assessment methods.			Secondary outcomes: Programme acceptability (understanding, interest, topic importance, likelihood of continued use), engagement with techniques, and take‐home exercise completion.		The changes in trait outcomes were more mixed. Some positive changes were observed, such as an increase in self‐compassion in the self‐compassion group, but the results were not uniformly positive across all measures.	
						Qualitative findings included feedback from both students and staff that informed the iterative development of the intervention materials. Students appreciated the relevance of the content but noted the need for more engaging video presentations and shorter lessons. Boys, in particular, found the content less relevant, which may have affected their engagement and acceptability ratings.	L: The study identified a difference in male and female student engagement which may indicate the need to review the content, delivery, or themes of the interventions to consider how to increase the appeal to males.
						Moderate adherence to programme protocol: Although the programme was video‐based, teachers facilitated the sessions and supported classroom delivery. The structure allowed for fair implementation but lacked manual adherence checks.	
O’Dea and Abraham (2000)	To evaluate the impact of an interactive, school‐based self‐esteem education program on the body image, eating attitudes, and behaviours of young male and female adolescents immediately following the program and after a 12‐month period.	Pre‐post intervention design, where participants were randomly assigned to either an intervention group that received a school‐based, self‐esteem education program or a control group that did not. Quantitative measures (e.g., body dissatisfaction, drive for thinness, self‐worth) and qualitative feedback from participant were utilised.	The sample featured *N* = 173 male and *N* = 297 female adolescents, with an average age of 13.0 years for males and 12.9 years for females. The participants were assessed on various metrics such as weight, height, body mass index (BMI), and standard body weight (SBW), as well as psychological metrics like drive for Thinness, body dissatisfaction, Global self‐worth, and anxiety and depression levels.	Primary outcomes: Body satisfaction, body image ratings, and eating attitudes (e.g., drive for thinness, body dissatisfaction, weight‐loss behaviours). Secondary outcomes: Self‐esteem dimensions (social acceptance, physical appearance, athletic competence), importance of close friendships, and perceptions of parental evaluation.	3 and 12 months	The intervention group experienced a significant decrease in mean body dissatisfaction subscale scores during the intervention (*F* = 8.6, *p* < 0.01), while the control group's scores increased. This improvement was not statistically significant at the 12‐month follow‐up.	S: The 12‐month follow‐up provided insights into the long‐term effects of the intervention, demonstrating that many of the positive effects were sustained.
						The intervention had a significant positive effect on high‐risk students, with their drive for Thinness scores decreasing (*F* = 4.0, *p* < 0.05) and significant improvement in physical appearance ratings, particularly. These improvements persisted at the 12‐month follow‐up but were not statistically significant.	
						Female students in the intervention group showed significant improvement in their self‐ratings and ratings they believed their parents would give them, particularly for father (*F* = 22.1, *p* < 0.001), which remained significant at the 12‐month follow‐up.	L: Much of the data, particularly on self‐esteem, body image, and eating behaviours gained from self‐report measures which could lead to social desirability bias, where students may report what they believe is expected rather than their true feelings or behaviours.
						There were no significant baseline differences in anthropometric measures between the groups. However, during the study period, the intervention group's standard body weight (SBW) increased significantly (*F* = 3.98, *p* < 0.05), particularly among normal‐weight females whose SBW increased (*F* = 5.53, *p* < 0.05), while the	
						The importance of social acceptance decreased significantly in the intervention group (*F* = 4.47, *p* < 0.05), an effect that persisted at the 12‐month follow‐up.	
						Athletic competence and physical appearance became less important for intervention students, especially those of low or high SBW. This was still the case after 12 months.	
						The number of female students in the intervention group who reported trying to lose weight increased significantly over the 12‐month period (*p* < 0.05), while the increase in the control group was not statistically significant.	
						High‐risk students (low self‐esteem, high anxiety) in the intervention group showed significant improvements in body dissatisfaction and drive for Thinness scores (*F* = 4.8, *p* < 0.05). The positive effects on body dissatisfaction persisted at the 12‐month follow‐up.	L: The study did not provide a detailed analysis of the costs involved in implementing the program versus the benefits gained. Understanding the cost‐effectiveness of such interventions is crucial for scaling them up in different educational settings.
						The majority of students commented that the program was to build self‐esteem and confidence rather than body image education.	
						89.6% of student participants stated that the program was valuable, citing improvements in self‐esteem, self‐image, and acceptance of diversity. A smaller percentage (10.4%) did not find the program valuable, often because they felt they already possessed the associated knowledge and that it did not develop their understanding further.	
						Around 70% of students expressed interest in participating in similar future programs. 50% of younger students (Year 7), reported positive changes in feelings, attitudes, and self‐concept, including increased confidence and better stress management. 95% of year 8 females a compared to 40% of males reported positive changes, such as improved self‐image, increased confidence, and greater tolerance for others.	
						High adherence to programme protocol: Teachers maintained close adherence to the intervention content. Follow‐up results demonstrated sustained outcomes, supporting fidelity.	
Phelps et al. (2000)	The study ‘an empirically supported eating disorder prevention program’ by Phelps et al. Aimed to develop and evaluate a six‐session prevention program designed to reduce risk factors and enhance protective factors associated with eating disorders among females in middle school, high school, and college.	The study used a pre‐post experimental‐control mixed methods design.	The sample for this study consisted of three groups of female participants:	Primary outcomes: Body dissatisfaction, drive for thinness, physical self‐concept, and personal competence.	Assessed immediately post‐intervention; No long‐term follow‐up was conducted, though booster sessions were recommended.	Middle school students: The assessment used included a scale developed by the senior researcher to measure. The program effectively reduced the future intended use of fasting, excessive exercise, purging, diet aids, water pills, and laxatives.	S: The study involved participants from middle school, high school, and college settings, allowing for the examination of the program's effectiveness across different age groups. This diversity enhances the generalisability.
			1. *N* = 530 female middle school students	Secondary outcomes: Attitudes toward sociocultural standards of beauty, current and future intentions to use disordered eating behaviours (fasting, purging, diet aids, excessive exercise), and program acceptability.		Decreases were observed in both body dissatisfaction and drive for thinness, although the changes did not reach statistical significance.	
						Improvements were seen in both physical self‐concept and competence, but similar to the other metrics, these increases were not statistically significant.	
			2. *N* = 312 female students			Teachers and students provided feedback indicating that at this age, very few of the female students were engaging in significant weight control activities or exhibiting disturbed eating behaviours. This feedback helped explain the lack of statistically significant results, as there was little room for measurable improvement within this young population.	L: The study focused mainly on assessing short‐term outcomes, with no long‐term follow‐up to evaluate whether the positive effects of the program were maintained over time. This lack of long‐term assessment limits the ability to determine the enduring effectiveness of the prevention efforts.
						High school students:	
	The study also aimed to provide a model for replication in other settings and suggested further research on the long‐term efficacy of the intervention.		High school students (159 of which were in the control group).			The experimental group showed a significant difference in overall Treatment efficacy scale scores compared to the control group (t [1, 310] = 22.01, *p* < 0.05).	L: Despite positive trends in reducing disordered eating behaviours and improving self‐concept, the study did not achieve statistically significant changes in most of the measured outcomes. This was attributed to the low baseline levels of disordered behaviours among participants.
						Body dissatisfaction and drive for Thinness both reduced, while physical self concept and competence scores increased. However, these changes, like those in the middle school group, did not reach statistical significance.	
			3. *N* = 45 female college students (of which 18 were in the control group). This 3rd group falls beyond the age requirement of this scoping review so will not be commented on.			The high school participants were less likely to engage in disordered eating behaviours, but similar to the middle school findings, the changes were not statistically significant.	
						High school students were more open to discussions and engaged more deeply in personal disclosure and group discussions. This resulted in positive qualitative feedback from participants and teachers, indicating greater participation and self‐disclosure compared to the middle school group.	
						Moderate adherence to programme protocol: Fidelity of implementation was unclear and not explicitly measured. The study relied on short‐term outcomes without confirming protocol adherence.	
Schmidt et al. (2017)	To design and evaluate the efficacy, feasibility, and acceptability of a teacher‐delivered universal prevention program, ‘Me, you & us,’ aimed at preventing eating disorders (EDs) among secondary school students. Additionally, the study aimed to assess the feasibility of training teachers to deliver the program and to evaluate how well the program was received by students across different school settings.	A cluster randomised controlled trial design where participant either accessed a six‐session teacher‐delivered body image intervention or a control group following the usual curriculum. Participants were assessed using questionnaires.	Students in years 8 and 9 from three state‐funded, all‐girls secondary schools in the UK. *N* = 261 students allocated to the intervention group and *N* = 187 students to the control group.	The primary outcome was body esteem, while secondary outcomes included binge eating, compensatory behaviours, thin‐ideal internalisation, appearance conversations with peers, peer support, depressive symptoms, self‐esteem, programme acceptability, and fidelity to the intervention. These measures assessed changes in participants' body image, eating behaviours, social influences, mental health, and perceptions of the programme.	3 months	Significant improvements in body esteem and reduced thin‐ideal internalisation among the intervention group compared to the control group were identified including at the 3 month follow up (body esteem: *d* = 0.19; thin‐ideal internalisation: *d* = 0.16).	S: The study not only assessed the efficacy of the intervention but also evaluated its feasibility and acceptability among both teachers and students, providing valuable insights for future implementation.
						However, the program did not produce significant differences between groups for other outcomes such as eating pathology, peer support, or depressive symptoms. Additionally, the acceptability and fidelity of the program varied across the three schools involved in the study.	L: With a smaller sample than planned, statistical power was 49% to identify group differences at follow up.
						Low adherence to programme protocol: The study reported that teachers often adapted or condensed the programme, deviating from the manualised format, which impacted effectiveness.	L: Variations in how closely teachers followed the intervention manual across different schools may have affected the program's effectiveness.
Sharpe et al. (2013)	To evaluate the suitability, practicality, and effectiveness of a body image intervention delivered by teachers.	A cluster randomised controlled trial design, where secondary school classes were randomly assigned to either receive a teacher‐delivered body image intervention or continue with the usual curriculum as a control. Participants, consisting of adolescents from UK secondary schools, were assessed using self‐report questionnaires at three time points: Pre‐intervention, post‐intervention, and 3‐month follow‐up. The intervention included six 50‐min lessons focused on media literacy, peer interactions, and positive psychology, with the primary outcomes measured being body esteem, thin‐ideal internalisation, and self‐esteem.	The sample in this study consisted of adolescent girls enrolled in three state‐funded UK secondary schools. A total of 448 students were initially involved, with 261 students in the intervention group and 187 students in the control group. The participants were drawn from years 8 and 9.	Primary outcomes: Body esteem.	Primary outcomes: Body esteem.	This teacher‐delivered body image intervention led to significant improvements in several key areas for the intervention group compared to the control group. Specifically:	S: The study included a sample from state‐funded schools with a range of ethnic background who received the interventions from their usual schoolteachers within a typical school setting, demonstrating the feasibility of this type of intervention.
					Secondary outcomes: Thin‐ideal internalisation, self‐esteem, binge eating, compensatory behaviours, appearance conversations with peers, peer support, depressive symptoms, and programme acceptability.	The intervention group showed a significant improvement in body esteem at the 3‐month follow‐up compared to the control group (*p* = 0.006), with an effect size of *d* = 0.19.	L: The consistency of the intervention's implementation varied across schools, with some teachers not fully following the intervention manual. This inconsistency may have influenced the intervention's effectiveness and limits the ability to draw definitive conclusions about its overall efficacy.
						A higher percentage of participants in the intervention group showed reliable improvement in body esteem (32%) compared to the control group (7%), a difference that was statistically significant (χ^2^ (1) = 5.97, *p* = 0.02).	
				Secondary outcomes: Thin‐ideal internalisation, self‐esteem, binge eating, compensatory behaviours, appearance conversations with peers, peer support, depressive symptoms, and programme acceptability.		The intervention group had significantly lower than‐ideal internalisation at both post‐intervention (*p* = 0.04) and at the 3‐month follow‐up (*p* = 0.05), with an effect size of *d* = 0.16 at follow‐up.	
					Follow‐up: Post‐intervention and at 3 months (underpowered)	There was a significant improvement in self‐esteem in the intervention group at post‐intervention (*p* = 0.04, *d* = 0.20). This was not found at the 3‐month follow‐up.	L: Classes rather than schools were used to randomise. Therefore, there is risk of contamination as students would not have been isolated from each other and could have discussed their experiences outside of the lessons with others. This limitation was however identified by the authors.
						No significant differences were found between the intervention and control groups for eating pathology or peer support.	
						High adherence to programme protocol: Teacher adherence was well documented and associated with significant and sustained improvements at follow‐up.	
Stewart et al. (2022)	Study 2: To evaluate the effectiveness of the ‘Happy being Me’ program in improving body satisfaction, addressing risk factors, enhancing self‐esteem, reducing symptoms of eating disorders, and increasing knowledge among students, To compare the outcomes between teacher‐led and clinician‐led groups to determine if there are any significant differences in effectiveness.	A non‐inferiority trial design to compare the effectiveness ‘Happy being Me’ when delivered by clinicians compared to teachers. Year 7 students from schools that had participated in an earlier study were involved, with outcomes assessed.	*N* = 346 Year 7 students from five schools with *N* = 174 students in the teacher‐led group and *N* = 172 in the clinician‐led group.	Primary outcome: Body satisfaction.	Follow‐up: Post‐intervention and at 3 months.	Body satisfaction significantly increased in the clinician‐led group but not in the teacher‐led group, with a medium‐sized effect maintained at follow‐up (*d* = 0.67).	S: The study utilised a robust non‐inferiority trial design, which is highly appropriate for comparing the effectiveness of two different intervention delivery methods (clinician‐led vs. teacher‐led).
		Outcome measures included:		Secondary outcomes: Internalisation of the thin ideal, physical appearance comparisons, appearance conversations, self‐esteem, eating disorder symptoms, and topic knowledge.		Both groups showed significant reductions in the internalisation of the thin body ideal and eating disorder symptoms, with small to medium effect sizes (*d* = 0.30 and *d* = 0.28, respectively), and improvements in self‐esteem (*d* = 0.20). Topic knowledge also increased significantly in both groups, although the clinician‐led group showed slightly higher scores, with a medium effect size (*d* = 0.35).	L: Teachers were able to modify the program according to their expertise and to fit the time they had to deliver. This could have weakened the interventions impact.
		Body satisfaction visual Analogue scale				There were no significant changes in physical comparison to others or appearance conversations over time.	L: The findings suggest that shifting the delivery of the program from clinicians to teachers might not achieve equivalent outcomes, particularly in key areas like body satisfaction. This raises questions about the effectiveness of task‐shifting in maintaining the impact of the intervention.
		Physical appearance comparison scale					
		Single‐item self‐esteem scale					
		A children's version of EAT.				Low adherence to programme protocol: Teachers made modifications to the programme based on time constraints or content preference, which may have weakened its intended effects.	
		Internalisation of the Thin ideal was measured via a subscale of the sociocultural attitudes Towards appearance questionnaire					
		The writers created a questionnaire to assess knowledge acquired from the program.					
Wade et al. (2003)	To compare the efficacy of a media literacy program and a self‐esteem program in reducing general and specific risk factors for eating disorders.	Controlled design to evaluate the effectiveness of two intervention programs of which participants were randomly assigned to one of these or a control group.	*N* = 86 Grade 8 students (*N* = 53 boys and 33 girls) from a private school in Australia.	Primary outcomes: Weight concern, shape concern, dietary restraint, and body dissatisfaction.	Post‐intervention and 3 months	At baseline, there were no significant differences between the groups in terms of variables or body mass index and no gender differences were found in self‐concept measures.	S: Unlike many studies, this research included both boys and girls, which is crucial for understanding the impact of the interventions across genders and addressing the risk factors that may affect them differently.
						Females exhibited significantly higher levels of weight concern (mean = 2.62, SD = 1.89) compared to boys (mean = 1.19, SD = 1.12), as well as higher levels of shape concern (mean = 2.78, SD = 1.73 for girls vs. mean = 1.50, SD = 1.42 for boys). While girls showed a higher degree of dietary restraint than boys, this difference was not statistically significant.	L: Due to time constraints, the intervention programs were condensed into five sessions, which may not have been sufficient to produce the desired intensity and impact of the interventions.
						Post‐intervention, the media literacy group showed a significant reduction in weight concern compared to the control group, with 79% reporting decreased weight concern, compared to 50% in the self‐esteem group and 29% in the control group.	L: Since different teachers, each with their unique approaches, delivered the media literacy and self‐esteem programs, determining whether the outcomes observed were a direct result of the program content or the way in which the material was presented is arduous.
				Secondary outcomes: Self‐esteem (subscales: Social acceptance, behavioural conduct, close friendships, self‐worth, scholastic competence).		No significant group differences were observed for body dissatisfaction or other specific risk factors at either post‐intervention or 3‐month follow‐up. At the 3‐month follow‐up, the media literacy group had higher perceived competence in close friendships, while the control group reported higher scholastic competence and behavioural conduct.	
		Standardised questionnaires were used for assessment.				Qualitative findings showed that students in the media literacy program commented that they now know about the hidden messages in advertising, the importance of being happy with oneself, and critical viewing skills.	
						Those in the self‐esteem group found stress management the most useful as well as the irrelevance of stereotypes, and self‐acceptance.t	
						Students were more engaged and interested in the media literacy program.	
						Moderate adherence to programme protocol: Although short in duration, the programme was delivered with consistency and adherence to the structure was generally maintained.	
Warschburger and Zitzmann (2018)	The aim of this study is to develop and evaluate a universal co‐educative prevention program, the POPS‐program (POtsdam prevention at schools), designed for adolescents in a school setting. The program seeks to reduce major risk factors associated with eating disorders, such as body dissatisfaction and unhealthy dieting, while promoting health through media literacy, strategies to resist social pressure, and the enhancement of coping skills and problem‐solving techniques.	A cluster‐randomised design involving six public high schools in potsdam, with three schools randomly assigned to the intervention group and three to a waiting control group. A range of questionnaires were used to gather data.	*N* = 1342 eligible participants aged 10–16 (*N* = 568 in the intervention group and *N* = 544 in the control group) from Germany	Primary outcomes: Disordered eating symptoms, bulimic symptoms, and drive for thinness.	3 and 12 months	There were significant group differences at baseline, with the intervention group showing higher body dissatisfaction compared to the control group.	S: A wide range of outcomes, including disordered eating behaviours, body dissatisfaction, internalisation of thin ideals, and other psychosocial factors, providing a detailed exploration.
				Secondary outcomes: Body dissatisfaction, internalisation of the thin ideal, perceived media pressure.		No significant baseline differences were observed for other variables.	L: The dropout rate was high with only 69.42% of the original baseline participants being able to be reviewed at the 1 year follow up. This was particularly the case with older students.
				Additional outcomes: Perfectionism, emotional element of exercise, social comparison, and perceived teasing.		Up to the 1 year follow up, those in the intervention group had reduced disordered eating symptoms and a highly significant decrease in body dissatisfaction after 3‐months	L: The study was conducted in a specific age group within the German school context which may limit the applicability of the findings to other age groups and countries.
						There were no significant interactions for bulimic symptoms or drive for thinness.	
						The intervention group had a significant difference in perceived media pressure over time—where the intervention group's values decreased and remained stable, while the control group's values increased.	
						Additionally, the intervention group showed a slight decrease in perfectionism and emotional attachment to exercise over a year, while these factors fluctuated or increased in the control group.	
						High adherence to programme protocol: Strong implementation fidelity was ensured through the use of a detailed manual and support structure, contributing to long‐term impact.	
Wolter et al. (2021)	To evaluate the efficacy and cost‐benefit of the ‘MaiStep’ universal school‐based prevention program	A cluster‐randomised controlled trial design involving 14 secondary schools, with classes randomly assigned to either the intervention group receiving the ‘MaiStep’ program, or the control group, which followed the standard curriculum.	*N* = 1654 students from grades 7 and 8 in German schools (average age = 13.4).	Primary outcome: DSM‐5 eating disorder diagnosis assessed using the SIAB‐S.	Post‐intervention and at 12 months.	The incidence of eating disorders was reduced. At post‐treatment, 6.1% of students in the active control group met the criteria for an eating disorder (6.6% at 12 months follow up), compared to only 3.2% (4% at 12 months follow up) in the intervention group.	S: The study effectively demonstrated that the MaiStep program reduced the incidence of eating disorders, with fewer participants in the intervention group meeting the DSM‐5 criteria for eating disorders at post‐treatment and at the 12‐month follow‐up compared to the control group.
		Evaluation occurred at baseline, post‐intervention, and 12‐months after a range of questionnaires measuring eating disorder symptoms, body image, and related psychological factors.	The sample consisted of 47.2% male and 52.8% female).	Secondary outcomes: Eating disorder risk factors, body dissatisfaction, and general health measures from self‐report questionnaires.		The program was also cost‐effective, saving approximately 560,000 euros per 1000 students by preventing 24 cases of eating disorders, demonstrating a benefit‐cost ratio of 6.75.	L: The cost‐benefit analysis was based on inflation‐adjusted data from older studies, which might not accurately reflect current healthcare costs.
						High adherence to programme protocol: The intervention was manualised and delivered as intended, with strong adherence confirmed by fidelity checks and sustained outcome measures.	L: Despite efforts to obtain informed consent from a large number of students, those who agreed to participate may differ systematically from those who did not, potentially introducing selection bias.
Yager et al. (2023)	To evaluate the effectiveness of the ‘Goodform’ program designed specifically for adolescent boys, to reduce body dissatisfaction and muscle‐building supplement use.	A cluster randomised controlled trial schools were randomised into either the intervention group, where they received the Goodform program, or a waitlist control group. Data collection occurred at baseline, post‐intervention, and an 8‐week follow‐up, using a variety of self‐report measures to assess body image, supplement use, and attitudes towards AAS.	*N* = 488 boys in grades 9 and 10 (mean age = 14.81) from nine public and independent, single sex and co‐educational secondary schools in Australia.	Primary outcomes: Muscularity dissatisfaction, body fat dissatisfaction, height dissatisfaction, attitudes toward anabolic steroid (AAS) use, intentions to use AAS, and use of muscle‐building supplements/AAS.	Post‐intervention (5 weeks) and at 8 weeks.	There were no significant interactions between the intervention condition and time across the 14 continuous outcome variables, but other notable main effects are:	S: The intervention was delivered by teachers during regular school hours, which may increase the feasibility and sustainability of the program in real‐world settings.
			Participants were randomly assigned to either the Goodform intervention group (*N* = 244) or the control group (*N* = 244).	Secondary outcomes: Negative body talk, internalisation of the muscular ideal, sociocultural pressures to be lean/muscular, and social norms around supplement and AAS use.		Boys in the intervention group reported lower levels of muscularity internalisation and less permissive social norms regarding anabolic androgenic steroid (AAS) use.	L: The participants' baseline level of muscle‐building supplement use was lower than anticipated, which might have constrained the detection of significant changes resulting from the intervention.
						In both the intervention and control group, there were significant increases in muscularity dissatisfaction, perceived pressure from significant others, peers, the media, and family, as well as increases in outcomes, expectancies, and intentions to use anabolic androgenic steroids. However, there was a significant decrease in muscularity internalisation over time.	L: The universal approach might not have been suitable for boys with initially low levels of body dissatisfaction or supplement use, which could have resulted in floor effects and an underestimation of the program's effectiveness.
						Overall, the intervention did not produce the expected outcomes, and in some cases, it was associated with more positive attitudes toward AAS use.	
						Low adherence to programme protocol: The implementation process was unclear. Limited information was available about teacher adherence, and outcome fidelity was weak.	

Across studies, short‐term improvements were more common than sustained outcomes, and the variability in outcome measures and follow‐up time points limits comparability. Nevertheless, programmes that reported stronger adherence to manualised delivery protocols tended to show greater impact, suggesting fidelity may play a key role in programme success (Table 3).

#### 3.3.2 Engagement and Feasibility

This review found that teachers can deliver programmes well with limited impact on the typical school day in a cost‐effective manner (Wade et al. 2003; Berger et al. 2014). When programmes can be adapted to suit the needs of the school setting and using teachers who have already built relationships with their students, the positive outcomes are far‐reaching due to the ease of facilitation reducing potential implementation barriers (Phelps et al. 2000).

Challenges were noted, particularly with regards to the consistent implementation and following protocols. Consequently, there are substantial differences in the reported fidelity of teacher‐led programmes due to several factors, including time demands requiring teachers to condense programmes and teachers changing content with the intention of better suiting classes (Sharpe et al. 2013).

Several studies reported non‐significant results. Phelps et al. (2000) observed decreases in body dissatisfaction and drive for thinness following the intervention, but changes remained statistically non‐significant at the immediate post‐intervention assessments. This lack of significance was likely due to low baseline levels of disordered behaviours. While the study refers to pre and post‐test comparisons, it did not specify the exact timing of the follow‐up assessments. Including clearly defined follow‐up time points would have been valuable for assessing delayed or sustained effects emerging over time, particularly as eating disorder risk may increase during later adolescence. Wade et al. (2003) found that though the media literacy program reduced weight concerns, there were no significant improvements in body dissatisfaction or other risk factors at either the immediate or 3‐month follow‐up. Favaro et al. (2005) identified no significant differences in BMI or overall Eating Attitudes Test scores between the intervention and control groups. Whilst Forbes et al. (2023) found improvements in self‐esteem and body esteem, this was not distinct from the control group. Yager et al. (2023) reported minimal impacts on muscularity dissatisfaction or body image concerns and, unexpectedly, even saw an increase in positive attitudes toward anabolic androgenic steroid use.

#### 3.3.3 Gender Differences

Media and self‐esteem programmes were more beneficial to females (Wade et al. 2003) whereas other studies found a reduction in male muscularity internalisation, and inconclusive findings were found with regards to supplement use (Yager et al. 2023).

## 4 Discussion

This review highlights potential effectiveness of teacher‐led interventions while revealing that there are some factors which can impede this.

### 4.1 The Role of Teachers in Eating Disorder Prevention

Teachers occupy a unique position within the school environment, spending substantial time with children and adolescents and thereby being well‐placed to observe changes in student wellbeing. This proximity enables them to play a significant role in both the prevention and early identification of eating disorders. The present review affirms that, when provided with appropriate training and support, teachers are capable of effectively delivering eating disorder prevention programmes. Evidence from studies such as Sharpe et al. (2013) and Wade et al. (2024) indicates that teacher‐led interventions can enhance self‐esteem, reduce thin‐ideal internalisation, and decrease both disordered eating behaviours and the incidence of eating disorders among young people (Favaro et al. 2005; Austin et al. 2007). For instance, Phelps et al. (2000) attributed positive outcomes to the low prevalence of weight control behaviours among female students at baseline.

The effectiveness of these interventions depends heavily on teacher adherence, with this review demonstrating that outcome variations often stem from differences in implementation. Schmidt et al. (2017), Stewart et al. (2022), and Forbes et al. (2023) have shown that when teachers diverge from the manualised program, program effectiveness is weakened. This may be attributed to teachers' proficiency in adapting content and learning structures to suit timetables and class abilities, an expected practice in this era of adaptive teaching. For instance, lesson elements deemed less essential are often omitted to save curriculum time. This reveals a core tension between the flexibility needed to accommodate professional judgement and classroom realities, and the requirement for fidelity to intervention protocols to ensure effectiveness. Future teacher‐led eating disorder prevention programmes must therefore stress the importance of maintaining core components. Clear guidance should differentiate between elements essential for fidelity and those open to contextual adaptation. This underscores the need for robust training and sustained support to promote consistent, high‐fidelity delivery. Strategies such as flexible implementation options and targeted resources may help teachers uphold fidelity while enhancing programme efficacy.

### 4.2 Gender‐Specific Considerations and the Role of Adherence

Inconsistency in the effectiveness of male and female students receiving teacher‐led interventions has been noted. It is plausible these discrepancies may be influenced by programme content and level of teacher adherence. Yager et al. (2023), Berger et al. (2014), Warschburger and Zitzmann (2018), and O'Dea and Abraham (2000) all highlight that many existing programmes are less effective for boys. This may be because the content does not fully reflect boys' lived experiences or priorities, making engagement with the material arduous. Teachers may also lack confidence in addressing the material with boys, given common stereotypes that eating disorders don't affect them, potentially explaining higher acceptability among girls reported by Kristoffersen et al. et al. (2022). This suggests a need for gender‐specific adaptations in content and delivery methods. Additionally, targeted teacher training to ensure they are equipped and appreciate that eating disorders can affect both genders, albeit with some differences in presentation.

### 4.3 Long‐Term Efficacy and Follow‐Up

Several studies, such as those by Atkinson et al. (2024) and Canetti et al. (2009), underscore the paramountcy of long‐term follow‐up to assess the sustainability of programme effects. While many programmes show favourable short‐term results, long‐term efficacy often depends on continued teacher adherence and reinforcement of the principles, which is methodologically challenging to assess. Wolter et al. (2021) demonstrate that consistent programme delivery can produce sustained reductions in eating disorder symptoms and improvements in body image over time. However, this is dependent on ongoing teacher engagement and fidelity to the programme, which in turn rely on strong support from school leadership and colleagues. A whole‐school approach, where prevention practices are continuously developed, is essential.

### 4.4 Cost‐Effectiveness and Feasibility

The review considers the cost‐effectiveness and feasibility of teacher‐led programmes, as highlighted by Wolter et al. (2021). It was found that teacher‐led programmes can be a cost‐effective means of delivering preventive interventions, especially in resource‐limited settings. However, the success of these programmes relies heavily on the training and ongoing support that teachers receive.

The distinction between clinician‐led and teacher‐led interventions is important in determining the most effective approach to school‐based eating disorder prevention. While both models offer distinct advantages/limitations, clinician‐led interventions benefit from specialised expertise and result in greater confidence and accuracy in programme delivery, which can positively influence outcomes.

They can provide more nuanced, therapeutic techniques, which have been found by Stewart et al. (2022) to achieve significant improvements in body satisfaction and reductions in symptoms associated with eating disorders. For example, the ‘Happy Being Me’ program, clinician‐led groups showed greater improvements in body satisfaction than teacher‐led groups, with effects sustained at follow‐up—suggesting that clinicians' expertise in delivering complex content can enhance outcomes.

The scalability, cost‐effectiveness, and sustainability of clinicians delivering school‐based interventions are limited in most countries. Budget constraints often prevent schools from funding such programmes, and clinical services are typically overstretched, making it difficult to release clinicians from their core duties. As a result, despite their potential for greater effectiveness, clinician‐led approaches face practical barriers that limit widespread implementation.

Conversely, teachers are already embedded in the educational system, allowing for more consistent and frequent delivery of interventions. Schmidt et al. (2017) and Forbes et al. (2023) demonstrated that teachers are adequately trained and adhere to the program protocols, they can achieve outcomes comparable to clinician‐led interventions, particularly in areas including thin‐ideal internalisation reduction. Teachers' continuing existence in students' lives and their established relationships allow them to reinforce the messages, fostering a supportive environment.

### 4.5 Implications for Policy and Practice

The findings of this scoping review have several important implications for policy and practice. There is a clear need for policies supporting the integration of mental health education, including eating disorder prevention, into school curriculum, so that teacher‐led interventions can benefit children and young people whilst not overburdening teachers. Schools should adopt a whole‐school approach to mental health, where eating disorder prevention is a crucial component.

A whole‐school approach can help prevent eating disorder interventions from being deprioritised amid competing mental health demands. Embedding programmes within a broader, coordinated strategy, supported by leadership, policies, and school culture, promotes consistent delivery and long‐term sustainability, positioning prevention as a core element of wellbeing rather than optional add‐ons.

Another point to consider is potential iatrogenic risks. Universal programmes may unintentionally heighten awareness or concern around body image and disordered eating, particularly among vulnerable or suggestible students. This possibility is supported by Yager et al. (2013), who highlighted the ethical dilemma of exposing low‐risk adolescents to material that could unintentionally normalise or prompt disordered thoughts or behaviours.

Furthermore, another important factor to consider is teacher adherence to program guidelines, which is critical to achieving desired outcomes. Schools must be supported in creating environments that promote positive body image and healthy eating behaviours. As part of this approach, teachers should receive ongoing professional development that reinforces the importance of adherence to program protocols. This could include regular refresher courses, peer support networks, and the provision of resources that make it easier for teachers to deliver the programmes as intended.

## 5 Conclusions and Future Directions

In conclusion, teachers play crucial roles in the prevention of eating disorders among adolescents. The evidence from this scoping review highlights potential effectiveness of teacher‐led interventions, it also urges the paramount importance of ensuring that teachers are well‐supported and adhere closely to intervention protocols, which is essential for maximising the effectiveness of programmes. By addressing challenges and building on strengths identified in the existing literature, schools can become pivotal environments for preventing eating disorders and promoting mental health among young people. As eating disorders increase, especially among adolescents, it is desirable that schools, supported by robust policies and evidence‐based practices, take an active role in prevention efforts.

Eating disorder prevention programmes should be integrated into the school curriculum to maximise reach, ensure regular reinforcement of key messages, and promote holistic approaches to student well‐being. Embedding these interventions within the broader educational framework also helps normalise conversations around body image and mental health. Given the increasing diversity of school populations, future research should focus on adapting existing programmes to be culturally sensitive and responsive to the needs of students from varied ethnic, neurodiverse, and gender backgrounds.

Additionally, more research is needed to examine the comparative effectiveness of clinician versus teacher‐led delivery models, particularly across diverse educational contexts. Such studies would help determine which approach is most effective in different settings and whether specific student groups benefit more from one particular model, guiding more equitable and targeted implementation strategies.

## Author Contributions

J.P. conceptualised the study with input from K.T. and K.A., J.P. conducted the initial literature review and J.P. and L.M. jointly reviewed papers. J.P. prepared the first draft of the manuscript. All authors reviewed and helped edit the final manuscript. Jessica Parker constructed and wrote the paper and conducted the initial data analysis. Lauren Makin scored selected papers and ensured that all papers fit the inclusion criteria. The review was shaped with the support of supervisors Professor Kate Tchanturia and Dr Karina Allen.

## Ethics Statement

Despite no participants being directly involved in this article, full ethical approval was gained via Kings College London's Research Ethics Committee.

## Consent

The authors have nothing to report.

## Conflicts of Interest

The authors declare no conflicts of interest.

## Data Availability

The datasets used and analysed during the current study are available from the corresponding author upon reasonable request.
